# Neoadjuvant Chemotherapy for Intrahepatic, Perihilar, and Distal Cholangiocarcinoma: a National Population-Based Comparative Cohort Study

**DOI:** 10.1007/s11605-023-05606-y

**Published:** 2023-02-07

**Authors:** Alessandro Parente, Sivesh K. Kamarajah, Marco Baia, Fabio Tirotta, Tommaso M. Manzia, Mohammed Abu Hilal, Timothy M. Pawlik, Steven A. White, Fadi S. Dahdaleh

**Affiliations:** 1 HPB and Transplant Unit, Department of Surgical Science, University of Rome Tor Vergata, Rome, Italy; 2 Department of Surgery, Queen Elizabeth Hospital Birmingham, University Hospital Birmingham NHS Trust, Birmingham, UK; 3 Academic Department of Surgery, Institute of Applied Health Research, University of Birmingham, Birmingham, UK; 4 Department of Surgery, Fondazione Poliambulanza - Istituto Ospedaliero, Brescia, Italy; 5 Division of Surgical Oncology, Department of Surgery, The Ohio State University Wexner Medical Center and James Comprehensive Cancer Center, Columbus, OH USA; 6 Department of HPB and Transplant Surgery, The Freeman Hospital, Newcastle Upon Tyne Hospitals NHS Foundation Trust, Newcastle University, Newcastle Upon Tyne, Newcastle, UK; 7 Edward-Elmhurst Health, Department of Surgical Oncology, Naperville, IL USA

**Keywords:** Perioperative chemotherapy, Outcomes, Cholangiocarcinoma, Resection, Survival

## Abstract

**Abstract:**

**Introduction:**

Data supporting the utilization of neoadjuvant chemotherapy (NAC) in patients receiving resection for cholangiocarcinoma (CCA) remains uncertain. We aimed to determine whether NAC followed by resection improves long-term survival in intrahepatic (iCCA), perihilar (hCCA), and distal (dCCA) cholangiocarcinoma, analyzed separately.

**Methods:**

Patients undergoing surgery for iCCA, hCCA, and dCCA, receiving either none, NAC, or adjuvant chemotherapy (AC) from 2010 to 2016 were identified from the National Cancer Database (NCDB). Cox regression was performed to account for selection bias and to assess the impact of surgery alone (SA) versus either NAC or AC on overall survival (OS).

**Results:**

There were 9411 patients undergoing surgery for iCCA (*n* = 3772, 39.5%), hCCA (*n* = 1879, 20%), and dCCA (*n* = 3760, 40%). Of these, 10.6% (*n* = 399), 6.5% (*n* = 123), and 7.2% (*n* = 271) with iCCA, hCCA, and dCCA received NAC, respectively. On adjusted analyses, patients receiving NAC followed by surgery had significantly improved OS, compared to SA for iCCA (*HR* 0.75, *CI*_95%_ 0.64–0.88, *p* < 0.001), hCCA (*HR* 0.72, *CI*_95%_ 0.54–0.97, *p* = 0.033), and for dCCA (*HR* 0.65, *CI*_95%_ 0.53–0.78, *p* < 0.001). However, sensitivity analyses demonstrated no differences in OS between NACs, followed by surgery or AC after surgery in iCCA (*HR* 1.19, *CI*_95%_ 0.99–1.45, *p* = 0.068), hCCA (*HR* 0.83 *CI*_95%_ 0.59–1.19, *p* = 0.311), and dCCA (*HR* 1.13 *CI*_95%_ 0.91–1.41, *p* = 0.264).

**Conclusions:**

This study associated NAC with increased OS for all CCA subtypes, even in patients with margin-negative and node-negative disease; however, no differences were found between NAC and AC. Our results highlight that a careful and interdisciplinary evaluation should be sought to consider NAC in CCA and warrant the need of larger studies to provide robust recommendation.

**Supplementary Information:**

The online version contains supplementary material available at 10.1007/s11605-023-05606-y.

## Introduction

Cholangiocarcinoma (CCA) is a rare, primary malignancy of the biliary system that arises from intra- and extrahepatic biliary tract epithelium. Anatomically, CCA are divided into intrahepatic CCA (iCCA), perihilar CCA (hCCA), and distal CCA (dCCA). Surgical resection remains the only potential for cure in localized disease; however, both local and distant relapses are common, even after complete resection.^1,2^ This has provided rationale to explore the role of adjuvant therapy, including radiation, chemoradiotherapy, and adjuvant chemotherapy (AC). The oncological benefits of AC have been corroborated in a number of studies and clinical trials^3–7^ and, today, AC is recommended in a majority of clinical guidelines.^8^

While neoadjuvant chemotherapy (NAC) has been linked to improved oncological outcomes in the setting of several gastrointestinal malignancies such as esophageal, gastric, and pancreatic cancer,^9–11^ the role of NAC in the management of localized CCA remains unclear.^12^ Existing evidence has largely been limited to retrospective studies and often examines NAC’s role prior to orthotopic liver transplantation.^13^ To date, comparable short- and long-term outcomes have been observed among resectable tumors that undergo upfront resection and advanced CCA treated with NAC, followed by surgery.^14–16^ Indeed, NAC may provide an advantage in the setting of large, locally advanced tumors with the goal of ultimate conversion to resectable disease.^14^ For example, in one study, NAC was associated with improved survival, compared to AC in CCA; however, stratification relative to CCA subtypes was not addressed separately.^17^

As such, the aim of this study was to investigate the role of NAC in iCCA, hCCA, and dCCA by performing a large, nationwide, high-quality, retrospective analysis study. Using contemporary data from the National Cancer Data Base (NCDB), the present sought to examine the association of NAC with survival among anatomical CCA subtypes, separately.

## Methods

### Data Source

The National Cancer Database (NCDB) is a joint project of the Commission on Cancer (CoC) of the American College of Surgeons and the American Cancer Society.^18^ Data from over 1500 CoC-accredited hospitals are collected to include > 70% of all newly diagnosed cancers in the USA. NCDB is a comprehensive of the large dataset, including details on demographics, facility type, and location, clinicopathologic tumor characteristics, type of treatment, and outcomes.

### Study Population

NCDB was used to identify patients > 18 years old diagnosed with non-metastatic iCCA, hCCA, and dCCA who underwent surgical resection between 2010 and 2016. International Classification of Disease for Oncology, Third Edition (ICD-O-3) was used to select adenocarcinoma and to exclude other histologies (ICD-O-3 morphology codes: 8240–8248). Patients were selected for inclusion in this study based on clinical stage; however, only patients who underwent formal resection were ultimately analyzed. Exclusion criteria were: (i) patients with concomitant cancer diagnoses, (ii) patients who underwent liver transplantation for CCA, (iii) gallbladder cancer, and (iv) missing data on receipt of perioperative therapy. Each CCA subtype was analyzed separately.

The following patient-level characteristics were analyzed: age (36–50, 51–65, 66–80, and > 80 years), race (white, black, and other), Charlson/Deyo comorbidity score (CDCC), year of diagnosis, insurance status (Medicaid/Medicare, Private Insurance, and Uninsured), zip code, level education status (i.e,. < 7.0%, 7.0%–12.9%, 13.0%–20.9%, and > 21.0%), nodal status (N0, N1, and N2), tumor grade/differentiation (well, moderate, poor, and anaplastic) and lymphovascular invasion (absent and present).

### Study Outcomes

Receipt of NAC was evaluated as the primary exposure variable. In the three cohorts which were analyzed separately, the overall survival (OS) was studied for between patients receiving surgery alone (SA) versus either NAC or AC and set as the primary outcome. OS was defined as the time between the date of CCA diagnosis and the date of death. Secondary outcomes were to compare OS in patients receiving NAC, followed by surgery or surgery followed by AC among the three subtypes of CCA, again analyzed separately.

### Statistical Analysis

Categorical variables were compared using the chi-squared test. Non-normally distributed data were analyzed using the Mann-Whitney *U* test. Survival was estimated using Kaplan-Meier survival curves and compared using the log-rank test. Multivariable analyses used binary logistic regression and Cox proportional hazard models adjusting for hospital-level (i.e., facility type and facility location), patient-level (age at diagnosis, sex, CDCC score, insurance status, education level, median income, and residence) and tumor-level (i.e., AJCC clinical T and clinical N stages) confounding factors. Sensitivity analyses were performed for each tumor subtypes, comparing only NAC with AC. A *p*-value of 0.05 was considered to be statistically significant throughout. Data analysis was performed using R Foundation Statistical software (R 3.2.2) with TableOne, ggplot2, Hmisc, Matchit, and survival packages (R Foundation for Statistical Computing, Vienna, Austria).

## Results

### Baseline Characteristics

Overall, there were 9411 patients who underwent surgery for iCCA (*n* = 3772, 39.5%), hCCA (*n* = 1879, 20%), and dCCA (*n* = 3760, 40%). Among the iCCA cohort, 399 (10.6%) patients received NAC, whereas 2140 (56.7%) underwent SA, and 1233 (32.7%) received surgery, followed by AC. A low proportion of patients diagnosed with hCCA received NAC (*n* = 123, 6.5%), as opposed to SA (*n* = 940, 50.0%) and AC (*n* = 816, 43.4%). Within the dCCA population, 271 (7.2%), 1953 (51.9%), and 1536 (40.9%), were treated with NAC with subsequent resection, SA and surgery followed by AC, respectively. Baseline patient and tumor characteristics of the three cohorts are summarized in Table 1 and supplementary Tables 1, 4, and 7. In addition, pathological characteristics are summarized in Table 3.

**Table 1:** Baseline characteristics of the three subtypes of CCA

Site of CCA	SA *n* = (%)	NAC *n* = (%)	AC *n* = (%)	*p*-value
Intrahepatic	*n* = 2140	*n* = 399	*n* = 1233	
Age at diagnosis				< 0.001
36–50	220 (10.3)	89 (22.3)	218 (17.7)	
51–65	778 (36.4)	192 (48.1)	552 (44.8)	
66–80	996 (46.5)	113 (28.3)	424 (34.4)	
80+	141 (6.6)	3 (0.8)	26 (2.1)	
Female	1071 (50.0)	192 (48.1)	671 (54.4)	0.021
CDCC				< 0.001
0–1	1897 (88.6)	368 (92.2)	1151 (93.3)	
2+	243 (11.4)	31 (7.8)	82 (6.7)	
Hilar	*n* = 940	*n* = 123	*n* = 816	
Age at diagnosis				< 0.001
36–50	69 (7.3)	22 (17.9)	101 (12.4)	
51–65	263 (28.0)	56 (45.5)	330 (40.4)	
66–80	491 (52.2)	41 (33.3)	361 (44.2)	
80+	114 (12.1)	2 (1.6)	24 (2.9)	
Female	350 (37.2)	52 (42.3)	324 (39.7)	0.394
CDCC				0.117
0–1	867 (92.2)	113 (91.9)	772 (94.6)	
2+	73 (7.8)	10 (8.1)	44 (5.4)	
Distal	*n* = 1953	*n* = 271	*n* = 1536	
Age at diagnosis				< 0.001
36–50	148 (7.6)	60 (22.1)	199 (13.0)	
51–65	634 (32.5)	126 (46.5)	644 (41.9)	
66–80	989 (50.6)	77 (28.4)	640 (41.7)	
80+	179 (9.2)	5 (1.8)	49 (3.2)	
Female	812 (41.6)	113 (41.7)	621 (40.4)	0.776
CDCC				< 0.001
0–1	1748 (89.5)	249 (91.9)	1442 (93.9)	
2+	205 (10.5)	22 (8.1)	94 (6.1)	

*CCA* cholangiocarcinoma, *CDCC* Charlson/Deyo comorbidity score

For iCCA, more patients who underwent surgery alone had a CDCC score of 2+ (11.4%), compared to those who had NAC or AC (7.8% and 6.7%, respectively, *p* < 0.001). Moreover, a larger proportion of iCCA patients treated with NAC underwent extended major resections: 72.9% (NAC) vs 48.8% (SA) and 54.7% (AC), *p* < 0.001. Conversely, a greater percentage of patients who underwent AC had positive margins: 33.9% (AC) vs 17.0% (SA) and 21.6% (NAC), *p* < 0.001. Otherwise, while those groups were statistically heterogeneous with respect to pathological T stage, N stage, and the presence of lymphovascular invasion, those differences were not clinically relevant (Supplementary Table 1). For hCCA, while no differences were noted in the extent of liver resection by treatment group, NAC was associated with lower rates of positive margins: 15.4% (NAC) vs 30.7% (SA) and 39.8% (AC), *p* > 0.001. Finally, for dCCA, a positive margin was more common in patients who received AC, whereas other relevant clinicopathological factors were relatively similar among treatment subgroups.

### iCCA

#### Overall Survival

For this cohort, the OS was 31.6 months (*CI*_95%_ 29.9–33.4 months) and 5-year survival 31% (Table 2). Among patients receiving AC, 35.4% (436/797) also received adjuvant radiation therapy, compared to only 4.5% (18/399) who received NAC (*p* < 0.001). NAC, compared to SA, was significantly associated with improved OS (*HR* 0.78, *CI*_95%_ 0.67–0.91, *p* = 0.001), and these results were confirmed after adjustment on multivariable model (*HR* 0.75, *CI*_95%_ 0.64–0.88, *p* < 0.001).

**Table 2:** Summary of impact of neoadjuvant and adjuvant chemotherapy on overall survival in patients with intrahepatic, hilar, and distal cholangiocarcinoma

	Patients	Overall survival, months	Adjusted *HR* (95% *CI*)*	*p*-value
iCCA				
SA	2140 (56.7)	31.4 (28.9–33.7)	REF	
NAC	399 (10.6)	37.6 (32.8–45.2)	0.75 (0.64–0.88)	< 0.001
AC	1233 (32.7)	29.9 (27.9–33.1)	0.85 (0.76–0.96)	0.008
hCCA				
SA	940 (50.0)	20.0 (18.3–22.8)	REF	
NAC	123 (6.5)	35.6 (27.3–50.6)	0.72 (0.54–0.97)	0.033
AC	816 (43.4)	29.2 (26.1–33-4)	0.63 (0.53–0.75)	< 0.001
dCCA				
SA	1953 (51.9)	21.8 (20.1–23.6)	REF	
NAC	271 (7.2)	38.1 (31.2–50.6)	0.65 (0.53–0.78)	< 0.001
AC	1536 (40.9)	28.0 (26.5–30.3)	0.73 (0.65–0.81)	< 0.001

*iCCA* intrahepatic cholangiocarcinoma, *hCCA* hilar/perihilar cholangiocarcinoma, *dCCA* distal cholangiocarcinoma

*Adjusted models include the following variables: facility type and location, hospital distance, year of diagnosis, age at diagnosis, gender, race, CDCC score, insurance status, education level, medical income, residence, surgery type, tumor grade, AJCC T and N stage, status of surgical margins, lymphovascular invasion, and adjuvant radiation therapy

#### Sensitivity Analyses

Sensitivity analyses were performed comparing patients receiving only NAC and AC to establish benefits between the two treatment options (Supplementary Tables 2 and 3). Median OS of patients receiving NAC were significantly higher than those receiving AC (median: 37.6 vs 29.9 months, respectively, *p* < 0.001). On adjusted analyses, although there was a trend towards higher benefits of NAC, this did not reach statistical significance, compared with AC at a multivariable model (*HR* 1.19 (0.99–1.45, *p* = 0.068) (Supplementary Table 3).

### hCCA

#### Overall Survival

Among patients with hCCA, the OS was 25.6 months (*CI*_95%_ 24.0–28.0 months) and 5-year survival 26% (Table 2). Negative margins were more frequent in patients receiving NAC (84.6%, *p* < 0.001). The majority of patients (*n* = 479, 58.7%) received adjuvant radiation therapy together with AC. The use of adjuvant radiation therapy was significantly lower in the SA (3.7%) and NAC (4.9%) population. NAC, compared to SA, was associated with improved OS at adjusted multivariable analysis (*HR* 0.72, *CI*_95%_ 0.54–0.97, *p* = 0.033).

#### Sensitivity Analyses

Sensitivity analyses were performed comparing patients receiving only NAC and AC to establish benefits between the two treatment options (Supplementary Table 5). Median OS of patients receiving NAC was higher than those receiving AC, without however reaching statistical significance (median: 35.6 vs 29.2, respectively, *p* = 0.4)*.* On adjusted analyses, there were no significant differences in outcomes between NAC and AC (*HR* 0.83, *CI*_95%_ 0.59–1.19, *p* = 0.311) (Supplementary Table 6).

### dCCA

#### Overall Survival

For this cohort, the OS was 25.8 months (*CI*_95%_ 24.4–37.0 months) and 5-year survival 36% (Table 2). Patients undergoing NAC had significantly higher rates of negative margins and absence of lymphovascular invasion (*p* < 0.001), as shown in Table 3. NAC, compared to SA, was significantly associated with improved OS after adjustment at multivariable model (*HR* 0.75, *CI*_95%_ 0.53–0.78, *p* < 0.001).

**Table 3:** Pathological characteristics of the study cohort

Site of CCA	SA *n* = (%)	NAC *n* = (%)	AC *n* = (%)	*p*-value
Intrahepatic	*n* = 2140	*n* = 399	*n* = 1233	
Tumor grade				< 0.001
Well	249 (21.6)	31 (7.8)	102 (8.3)	
Moderate	1072 (50.1)	138 (34.6)	593 (48.1)	
Poor	529 (24.7)	101 (25.3)	387 (31.4)	
Anaplastic	290 (13.6)	129 (32.3)	151 (12.2)	
AJCC pathological T stage				< 0.001
pTx	592 (27.7)	131 (32.8)	280 (22.7)	
pT1	665 (31.1)	121 (30.3)	208 (16.9)	
pT2	540 (25.2)	81 (20.3)	414 (33.6)	
pT3	232 (10.8)	43 (10.8)	224 (18.2)	
pT4	111 (5.2)	23 (5.8)	107 (8.7	
AJCC pathological N stage				< 0.001
N0	906 (42.3)	173 (43.4)	486 (39.4)	
N1	273 (12.8)	61 (15.3)	314 (25.5)	
N2	47 (2.2)	9 (2.3)	54 (4.4)	
N3	8 (0.4)	2 (0.5)	24 (1.9)	
Nx	906 (42.3)	154 (38.6)	355 (28.8)	
Margin negative	1777 (83.0)	313 (78.4)	815 (66.1)	< 0.001
Lymphovascular absent	1715 (80.1)	338 (84.7)	830 (67.3)	< 0.001
Adjuvant RT	51 (2.4%)	18 (4.5)	436 (35.4)	< 0.001
Hilar	*n* = 940	*n* = 123	*n* = 816	
Tumor grade				< 0.001
Well	140 (14.9)	13 (10.6)	99 (12.1)	
Moderate	402 (42.8)	25 (20.3)	404 (49.5)	
Poor	248 (26.4)	16 (13)	121 (26)	
Anaplastic	150 (16)	69 (56.1)	101 (12.4)	
AJCC pathological T stage				< 0.001
pTx	86 (9.1)	28 (22.8)	52 (6.4)	
pT1	164 (17.4)	22 (17.9)	41 (5)	
pT2	451 (48)	43 (35)	441 (54)	
pT3	197 (21)	17 (13.8)	236 (28.9)	
pT4	42 (4.5)	13 (10.6)	46 (5.6)	
AJCC pathological N stage				< 0.001
N0	493 (52.4)	69 (56.1)	320 (39.2)	
N1	239 (25.4)	16 (13)	302 (37)	
N2	39 (4.1)	6 (4.9)	64 (7.8)	
N3	14 (1.5)	1 (0.8)	27 (3.3)	
Nx	155 (16.5)	31 (25.2)	103 (12.6)	
Margin negative	651 (69.3)	104 (84.6)	491 (60.2)	< 0.001
Lymphovascular absent	637 (67.8)	100 (81.3)	505 (61.9)	< 0.001
Adjuvant RT	35 (3.7)	6 (4.9)	479 (58.7)	< 0.001
Distal	*n* = 1953	*n* = 271	*n* = 1536	
Tumor grade				< 0.001
Well	252 (12.9)	26 (9.6)	123 (8)	
Moderate	887 (45.4)	75 (27.7)	714 (46.5)	
Poor	532 (27.2)	53 (19.6)	493 (32.1)	
Anaplastic	282 (14.4)	117 (43.2)	206 (13.4)	
AJCC pathological T stage				< 0.001
pTx	638 (32.7)	103 (38)	351 (22.9)	
pT1	281 (14.4)	58 (21.4)	73 (4.8)	
pT2	498 (25.5)	47 (17.3)	461 (30)	
pT3	465 (23.8)	47 (17.3)	579 (37.7)	
pT4	71 (3.6)	16 (5.9)	72 (4.7)	
AJCC pathological N stage				< 0.001
N0	930 (47.6)	136 (50.2)	599 (39)	
N1	407 (20.8)	45 (16.6)	539 (35.1)	
N2	87 (4.5)	7 (2.6)	119 (7.7)	
N3	34 (1.78)	4 (1.5)	66 (4.3)	
Nx	495 (25.3)	79 (29.2)	213 (13.9)	
Margin negative	1517 (77.7)	219 (80.8)	1033 (67.3)	< 0.001
Lymphovascular absent	1592 (81.5)	234 (86.3)	1086 (70.7)	< 0.001
Adjuvant RT	71 (3.6)	22 (8.1)	814 (53.0)	< 0.001

#### Sensitivity Analyses

Sensitivity analyses were performed comparing patients receiving only NAC and AC to establish benefits between the two treatment options (Supplementary Table 8). Median OS of patients receiving NAC were significantly higher than those receiving AC (median: 38.1 vs 28 months, *p* < 0.001*).* On adjusted analyses, there were no significant differences in outcomes between NAC and AC (*HR* 1.13, *CI*_95%_ 0.91–1.41, *p* = 0.264) (Supplementary Table 9).

## Discussion

The role of neoadjuvant chemotherapy in patients with cholangiocarcinoma (CCA) remains unclear owing to a lack of prospective or large studies to date. The present study, which examined 9411 patients, found that neoadjuvant chemotherapy (NAC) versus surgery alone was associated with improved OS for CCA when stratified by anatomical location. Moreover, while NAC compared to AC was similarly associated with increased OS, this effect did not persist after performing sensitivity analyses among patients treated with NAC and AC among all three subtypes of CCA. Findings from this study suggest that overall compliance, rather than sequence, of multimodality therapy may impact OS for CCA anatomical subgroups and support the potential role for NAC in the management of this disease.

**Fig. 1:**
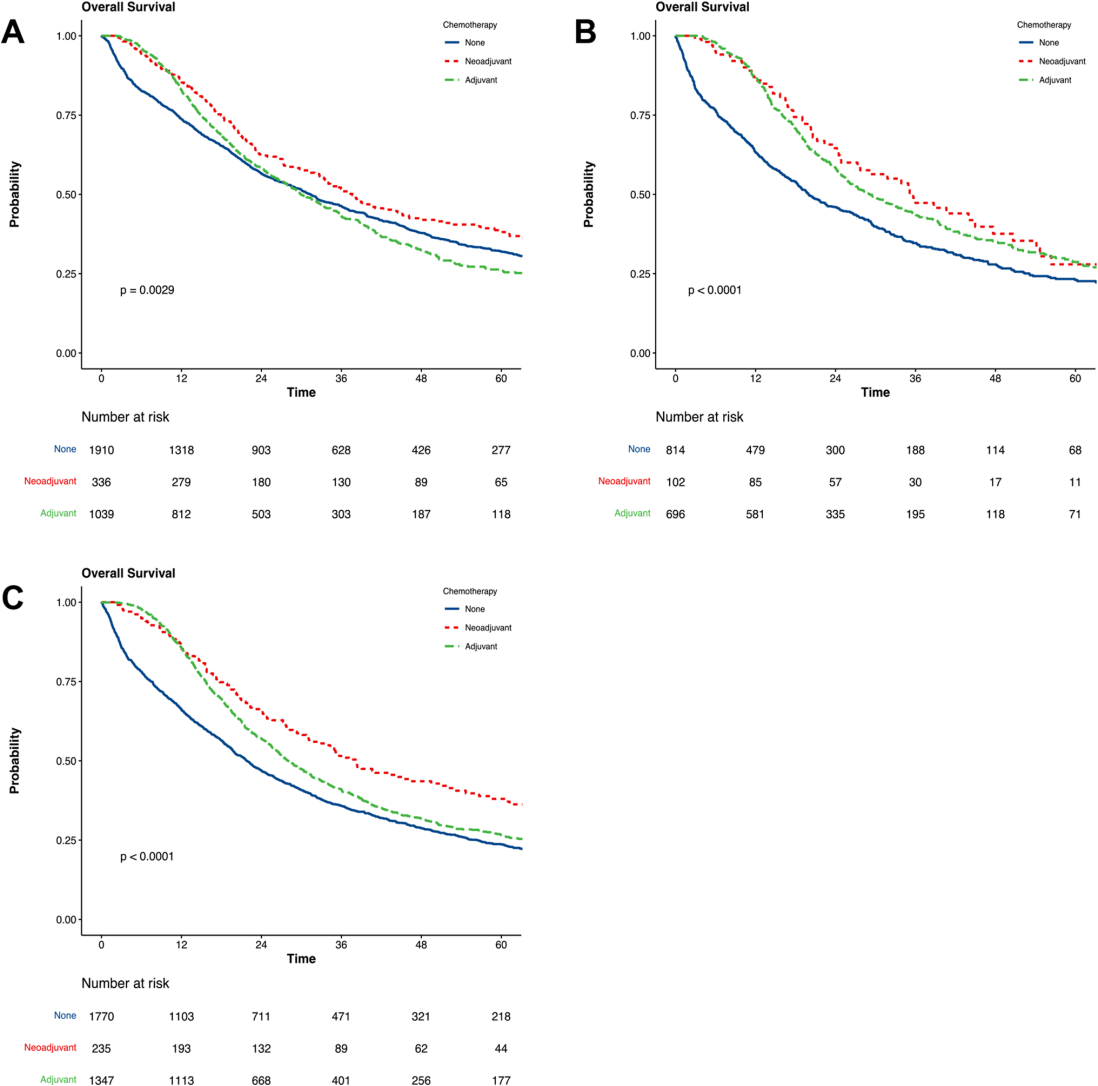
Impact of neoadjuvant and adjuvant chemotherapy on long-term survival after resection for cholangiocarcinoma **A** Intrahepatic. **B** Hilar. **C** Distal

NAC represents an appealing approach in oncology, as it can potentially control disease progression, reduce tumor volume, increase R0 resection rates, and possibly avoid non-curative resections among patients who progress on systemic therapy. Although randomized trials are ongoing, to date there is a lack of robust data evaluating NAC’s role in the management of CCA, as the majority of the literature is derived from small retrospective studies.^11,12,16^ For example, a single-center study of 74 patients with locally advanced iCCA has demonstrated that treatment with NAC, followed by surgery, provided similar short- and long-term results, compared to patients with initially resectable ICC who had upfront surgery.^16^ This suggests that NAC may be utilized as a first-line treatment for locally advanced iCCA in an effort to downstage locally advanced disease and increased likelihood of resectability. Results from this report are consistent with these findings, as iCCA patients treated with NAC, followed by surgery achieved improved OS, compared to upfront resection or resection followed by AC (*p* = 0.0029; Fig. 1). In this regard, it is important to underline that the decision for different treatments is tailored for each patient based both on the clinical stage and clinical conditions, rather than the pathological state alone. Recently, a report from NCBD similarly linked NAC with longer OS in a select group of patients with CCA when compared to upfront resection, followed by AC.^17^ The present study is different, as it uses a more contemporary edition of NCDB and further stratifies CCA anatomically into iCCA, hCCA, and dCCA, and we have analyzed all the subtypes separately.

Interestingly, after sensitivity analyses, OS was comparable among patients receiving NAC or AC in this study. These findings signal that compliance with multimodal therapy, which includes surgery and systemic therapy, rather than sequencing may affect the oncologic outcomes we observed in this retrospective study. Importantly, AC and NAC patients are likely heterogeneous. First, patients selected for upfront surgery likely had anatomically resectable cancers at diagnosis, whereas NAC patients were more likely to include locally advanced or borderline resectable tumors. As guidelines do not uniformly recommend NAC for CCA, NAC may represent a viable approach in the management of locally advanced CCAs, irrespective of anatomical subtype. Second, it is possible that NAC patients were perceived to have poor performance status precluding upfront surgery. Similarly, given comparable OS for AC and NAC, NAC may be considered in circumstances where rehabilitation prior to surgery is possible. In addition, patients who underwent NAC and had subsequent disease progression will not be included inherently in the NCDB and hence excluded from this analysis. Last, as patients who underwent NAC and had subsequent disease progression were not included in this analysis, it is likely that NAC is a useful tool to select for more favorable biology and ultimately avoid non-curative resections. While the lack of important granular data on disease progression and treatment selection rationale may have led to selection bias, it is not possible to determine to what degree competing biases affected outcomes in this report.

The retrospective nature of this study and inherent limitations with the NCDB database limits the conclusions that we can draw from these results. First, despite attempting to statically correct for confounders through multivariable and sensitivity analyses, treatment selection bias may not have been entirely accounted for. Specifically, patients selected for surgery first and those who remained eligible to receive AC were likely better overall performers and therefore had improved OS. As NAC arguably also selected for better tumor biology, it remains unclear how sequence impacts survival in this setting. Second, important granular details including type of chemotherapy treatment regimen, duration, dosage, and response are missing in NCDB. In this regard, differences in chemotherapeutic regimens could have played a considerable role when interpreting outcomes. Also, it was not possible to compare the regimen of perioperative chemotherapy (NAC plus AC) in the three CCA subgroups, given the small number of patients who received such treatment. This would have provided a better understanding of the role of perioperative chemotherapy in CCA. Third, data on mode of recurrences and disease-free survival are missing from this dataset which further limits interpretation. Fourth, the exact causes of death are not reported on NCBD, and this could potentially lead to bias when calculating OS, as death may be related to side effects of chemotherapy, surgical complications, or other factors without being directly related only to the disease itself. Fifth, included in the multivariable regression model were factors which may have been affected by NAC such as margin status and tumor grade. As NCDB does not include details on pathological response to NAC, it is possible those factors were artificially improved for patients receiving upfront therapy thereby diminishing their true effect on OS. Moreover, age was treated as a categorical variable, and it is possible that selected cutoffs did not coincide with measurable effects. Finally, as NCBD includes only Commission on Cancer Hospitals, our findings might not be generalizable to broader population or other community hospitals.

## Conclusion

In summary, notwithstanding several limitations, in this retrospective study which used a contemporary national dataset, we found that NAC, followed by surgery for iCCA, hCCA, and dCCA, was associated with increased survival, compared with SA, regardless of nodal or margin status. While an incremental advantage of NAC, compared to AC was not observed on sensitivity analysis, those results highlight that careful and interdisciplinary evaluation should incorporate NAC in the management of CCA and warrant the need of large multicenter studies or randomized trials to refine its role.

## Supplementary Information

ESM 1 (DOCX 47 kb)

## Abbreviations

AC: adjuvant chemotherapy
dCCA: distal cholangiocarcinoma
iCCA: intrahepatic cholangiocarcinoma
hCCA: perihilar cholangiocarcinoma
NAC: neoadjuvant chemotherapy
SA: surgery alone
