# Supplementary material for: Neoadjuvant Chemotherapy for Intrahepatic, Perihilar, and Distal Cholangiocarcinoma: a National Population-Based Comparative Cohort Study
Source: J Gastrointest Surg. 2023 Feb 7;27(4):741–9. doi: 10.1007/s11605-023-05606-y (PMC10073049; doi:10.1007/s11605-023-05606-y)
Supplement: Supplementary file 1 — (DOCX 47 kb) [file 11605_2023_5606_MOESM1_ESM.docx]

Supplementary Table 1. Characteristics of patients undergoing SA, NAC or AC for intrahepatic cholangiocarcinoma.

|  |  | Surgery Alone | Neoadjuvant | Adjuvant | P value |
| --- | --- | --- | --- | --- | --- |
| Facility Type | Community | 428 (20.0) | 55 (13.8) | 315 (25.5) | <0.001 |
|  | Academic | 1387 (64.8) | 273 (68.4) | 723 (58.6) |  |
|  | Others | 325 (15.2) | 71 (17.8) | 195 (15.8) |  |
| Facility Location | Northeast | 448 (20.9) | 64 (16.0) | 349 (28.3) | <0.001 |
|  | South | 785 (36.7) | 127 (31.8) | 412 (33.4) |  |
|  | Midwest | 590 (27.6) | 124 (31.1) | 255 (20.7) |  |
|  | West | 317 (14.8) | 84 (21.1) | 217 (17.6) |  |
| Hospital Distance | <12.5 miles | 717 (33.5) | 95 (23.8) | 543 (44.0) | <0.001 |
|  | 12.5-49.9 miles | 698 (32.6) | 140 (35.1) | 438 (35.5) |  |
|  | >/=50 miles | 725 (33.9) | 164 (41.1) | 252 (20.4) |  |
| Year of Diagnosis | 2006-2007 | 272 (12.7) | 36 (9.0) | 107 (8.7) | <0.001 |
|  | 2008-2009 | 343 (16.0) | 57 (14.3) | 168 (13.6) |  |
|  | 2010-2011 | 392 (18.3) | 71 (17.8) | 199 (16.1) |  |
|  | 2012-2013 | 426 (19.9) | 86 (21.6) | 238 (19.3) |  |
|  | 2014-2016 | 707 (33.0) | 149 (37.3) | 521 (42.3) |  |
| Age at Diagnosis, years | 36-50 | 220 (10.3) | 89 (22.3) | 218 (17.7) | <0.001 |
|  | 51-65 | 778 (36.4) | 192 (48.1) | 552 (44.8) |  |
|  | 66-80 | 996 (46.5) | 113 (28.3) | 424 (34.4) |  |
|  | 80+ | 141 (6.6) | 3 (0.8) | 26 (2.1) |  |
|  | (Missing) | 5 (0.2) | 2 (0.5) | 13 (1.1) |  |
| Sex | Male | 1069 (50.0) | 207 (51.9) | 562 (45.6) | 0.021 |
|  | Female | 1071 (50.0) | 192 (48.1) | 671 (54.4) |  |
| Race | White | 1824 (85.2) | 358 (89.7) | 1042 (84.5) | 0.031 |
|  | Black | 161 (7.5) | 18 (4.5) | 81 (6.6) |  |
|  | Other | 155 (7.2) | 23 (5.8) | 110 (8.9) |  |
| CDCC Score | 0-1 | 1897 (88.6) | 368 (92.2) | 1151 (93.3) | <0.001 |
|  | 2+ | 243 (11.4) | 31 (7.8) | 82 (6.7) |  |
| Insurance Status | Uninsured | 147 (6.9) | 17 (4.3) | 63 (5.1) | <0.001 |
|  | Private Insurance | 759 (35.5) | 225 (56.4) | 649 (52.6) |  |
|  | Medicaid | 105 (4.9) | 21 (5.3) | 56 (4.5) |  |
|  | Medicare | 1129 (52.8) | 136 (34.1) | 465 (37.7) |  |
| Education Level | >21% | 363 (17.0) | 45 (11.3) | 173 (14.0) | <0.001 |
|  | 13%-20.9% | 509 (23.8) | 72 (18.0) | 269 (21.8) |  |
|  | 7%-12.9% | 693 (32.4) | 144 (36.1) | 396 (32.1) |  |
|  | <7% | 575 (26.9) | 138 (34.6) | 395 (32.0) |  |
| Medical Income | </=$47,999 | 841 (39.3) | 133 (33.3) | 392 (31.8) | <0.001 |
|  | $48,000-$62,999 | 554 (25.9) | 111 (27.8) | 317 (25.7) |  |
|  | $63,000 + | 745 (34.8) | 155 (38.8) | 524 (42.5) |  |
| Residence | Metro | 1717 (80.2) | 327 (82.0) | 1023 (83.0) | 0.076 |
|  | Urban | 317 (14.8) | 61 (15.3) | 151 (12.2) |  |
|  | Rural | 106 (5.0) | 11 (2.8) | 59 (4.8) |  |
| Surgery Type | Major Hepatectomy | 115 (5.4) | 13 (3.3) | 49 (4.0) | <0.001 |
|  | 36 | 465 (21.7) | 47 (11.8) | 215 (17.4) |  |
|  | 37 | 483 (22.6) | 40 (10.0) | 266 (21.6) |  |
|  | 38 | 32 (1.5) | 8 (2.0) | 29 (2.4) |  |
|  | Extended Major Hepatectomy | 1045 (48.8) | 291 (72.9) | 674 (54.7) |  |
| Tumour Grade | Well | 249 (11.6) | 31 (7.8) | 102 (8.3) | <0.001 |
|  | Moderate | 1072 (50.1) | 138 (34.6) | 593 (48.1) |  |
|  | Poor | 529 (24.7) | 101 (25.3) | 387 (31.4) |  |
|  | Anaplastic | 290 (13.6) | 129 (32.3) | 151 (12.2) |  |
| AJCC Pathological T Stage | pTx | 592 (27.7) | 131 (32.8) | 280 (22.7) | <0.001 |
|  | pT1 | 665 (31.1) | 121 (30.3) | 208 (16.9) |  |
|  | pT2 | 540 (25.2) | 81 (20.3) | 414 (33.6) |  |
|  | pT3 | 232 (10.8) | 43 (10.8) | 224 (18.2) |  |
|  | pT4 | 111 (5.2) | 23 (5.8) | 107 (8.7) |  |
| AJCC Pathological N Stage | N0 | 906 (42.3) | 173 (43.4) | 486 (39.4) | <0.001 |
|  | N1 | 273 (12.8) | 61 (15.3) | 314 (25.5) |  |
|  | N2 | 47 (2.2) | 9 (2.3) | 54 (4.4) |  |
|  | N3 | 8 (0.4) | 2 (0.5) | 24 (1.9) |  |
|  | Nx | 906 (42.3) | 154 (38.6) | 355 (28.8) |  |
| Margin Status | Negative | 1777 (83.0) | 313 (78.4) | 815 (66.1) | <0.001 |
|  | Positive | 363 (17.0) | 86 (21.6) | 418 (33.9) |  |
| Lymphovascular Invasion | Absent | 1715 (80.1) | 338 (84.7) | 830 (67.3) | <0.001 |
|  | Present | 425 (19.9) | 61 (15.3) | 403 (32.7) |  |
| Adjuvant Radiotherapy | No | 2089 (97.6) | 381 (95.5) | 797 (64.6) | <0.001 |
|  | Yes | 51 (2.4) | 18 (4.5) | 436 (35.4) |  |

Supplementary Table 2. Characteristics among patient undergoing NAC and AC for iCCA.

|  |  | Neoadjuvant | Adjuvant | P value |
| --- | --- | --- | --- | --- |
| Facility Type | Community | 55 (13.8) | 315 (25.5) | <0.001 |
|  | Academic | 273 (68.4) | 723 (58.6) |  |
|  | Others | 71 (17.8) | 195 (15.8) |  |
| Facility Location | Northeast | 64 (16.0) | 349 (28.3) | <0.001 |
|  | South | 127 (31.8) | 412 (33.4) |  |
|  | Midwest | 124 (31.1) | 255 (20.7) |  |
|  | West | 84 (21.1) | 217 (17.6) |  |
| Hospital Distance | <12.5 miles | 95 (23.8) | 543 (44.0) | <0.001 |
|  | 12.5-49.9 miles | 140 (35.1) | 438 (35.5) |  |
|  | >/=50 miles | 164 (41.1) | 252 (20.4) |  |
| Year of Diagnosis | 2006-2007 | 36 (9.0) | 107 (8.7) | 0.529 |
|  | 2008-2009 | 57 (14.3) | 168 (13.6) |  |
|  | 2010-2011 | 71 (17.8) | 199 (16.1) |  |
|  | 2012-2013 | 86 (21.6) | 238 (19.3) |  |
|  | 2014-2016 | 149 (37.3) | 521 (42.3) |  |
| Age at Diagnosis, years | 36-50 | 89 (22.3) | 218 (17.7) | 0.014 |
|  | 51-65 | 192 (48.1) | 552 (44.8) |  |
|  | 66-80 | 113 (28.3) | 424 (34.4) |  |
|  | 80+ | 3 (0.8) | 26 (2.1) |  |
|  | (Missing) | 2 (0.5) | 13 (1.1) |  |
| Sex | Male | 207 (51.9) | 562 (45.6) | 0.033 |
|  | Female | 192 (48.1) | 671 (54.4) |  |
| Race | White | 358 (89.7) | 1042 (84.5) | 0.034 |
|  | Black | 18 (4.5) | 81 (6.6) |  |
|  | Other | 23 (5.8) | 110 (8.9) |  |
| CDCC Score | 0-1 | 368 (92.2) | 1151 (93.3) | 0.514 |
|  | 2+ | 31 (7.8) | 82 (6.7) |  |
| Insurance Status | Uninsured | 17 (4.3) | 63 (5.1) | 0.449 |
|  | Private Insurance | 225 (56.4) | 649 (52.6) |  |
|  | Medicaid | 21 (5.3) | 56 (4.5) |  |
|  | Medicare | 136 (34.1) | 465 (37.7) |  |
| Education Level | >21% | 45 (11.3) | 173 (14.0) | 0.122 |
|  | 13%-20.9% | 72 (18.0) | 269 (21.8) |  |
|  | 7%-12.9% | 144 (36.1) | 396 (32.1) |  |
|  | <7% | 138 (34.6) | 395 (32.0) |  |
| Medical Income | </=$47,999 | 133 (33.3) | 392 (31.8) | 0.427 |
|  | $48,000-$62,999 | 111 (27.8) | 317 (25.7) |  |
|  | $63,000 + | 155 (38.8) | 524 (42.5) |  |
| Residence | Metro | 327 (82.0) | 1023 (83.0) | 0.079 |
|  | Urban | 61 (15.3) | 151 (12.2) |  |
|  | Rural | 11 (2.8) | 59 (4.8) |  |
| Surgery Type | Major Hepatectomy | 13 (3.3) | 49 (4.0) | <0.001 |
|  | 36 | 47 (11.8) | 215 (17.4) |  |
|  | 37 | 40 (10.0) | 266 (21.6) |  |
|  | 38 | 8 (2.0) | 29 (2.4) |  |
|  | Extended Major Hepatectomy | 291 (72.9) | 674 (54.7) |  |
| Tumour Grade | Well | 31 (7.8) | 102 (8.3) | <0.001 |
|  | Moderate | 138 (34.6) | 593 (48.1) |  |
|  | Poor | 101 (25.3) | 387 (31.4) |  |
|  | Anaplastic | 129 (32.3) | 151 (12.2) |  |
| AJCC Pathological T Stage | pTx | 131 (32.8) | 280 (22.7) | <0.001 |
|  | pT1 | 121 (30.3) | 208 (16.9) |  |
|  | pT2 | 81 (20.3) | 414 (33.6) |  |
|  | pT3 | 43 (10.8) | 224 (18.2) |  |
|  | pT4 | 23 (5.8) | 107 (8.7) |  |
| AJCC Pathological N Stage | N0 | 173 (43.4) | 486 (39.4) | <0.001 |
|  | N1 | 61 (15.3) | 314 (25.5) |  |
|  | N2 | 9 (2.3) | 54 (4.4) |  |
|  | N3 | 2 (0.5) | 24 (1.9) |  |
|  | Nx | 154 (38.6) | 355 (28.8) |  |
| Margin Status | Negative | 313 (78.4) | 815 (66.1) | <0.001 |
|  | Positive | 86 (21.6) | 418 (33.9) |  |
| Lymphovascular Invasion | Absent | 338 (84.7) | 830 (67.3) | <0.001 |
|  | Present | 61 (15.3) | 403 (32.7) |  |
| Adjuvant Radiotherapy | No | 381 (95.5) | 797 (64.6) | <0.001 |
|  | Yes | 18 (4.5) | 436 (35.4) |  |

Supplementary Table 3. Cox regression multivariable model on overall survival for intrahepatic cholangiocarcinoma

|  |  | Hazard ratio (CI_95%_) | P value |
| --- | --- | --- | --- |
| Facility Type | Community | REF |  |
|  | Academic | 0.75 (0.62-0.90) | 0.003 |
|  | Others | 0.88 (0.70-1.11) | 0.283 |
| Facility Location | Northeast | REF |  |
|  | South | 1.30 (1.08-1.58) | 0.007 |
|  | Midwest | 1.09 (0.88-1.34) | 0.443 |
|  | West | 0.95 (0.74-1.21) | 0.675 |
| Hospital Distance | <12.5 miles | REF |  |
|  | 12.5-49.9 miles | 1.34 (1.14-1.59) | 0.001 |
|  | >/=50 miles | 1.16 (0.93-1.45) | 0.181 |
| Year of Diagnosis | 2006-2007 | REF |  |
|  | 2008-2009 | 0.88 (0.69-1.13) | 0.330 |
|  | 2010-2011 | 0.77 (0.59-0.99) | 0.044 |
|  | 2012-2013 | 0.73 (0.57-0.95) | 0.017 |
|  | 2014-2016 | 0.68 (0.51-0.89) | 0.005 |
| Age at Diagnosis, years | 36-50 | REF |  |
|  | 51-65 | 1.12 (0.92-1.37) | 0.270 |
|  | 66-80 | 1.65 (1.26-2.16) | <0.001 |
|  | 80+ | 1.52 (0.82-2.81) | 0.188 |
| Sex | Male | REF |  |
|  | Female | 0.69 (0.60-0.79) | <0.001 |
| Race | White | REF |  |
|  | Black | 1.13 (0.83-1.54) | 0.439 |
|  | Other | 0.88 (0.67-1.15) | 0.342 |
| CDCC Score | 0-1 | REF |  |
|  | 2+ | 1.11 (0.83-1.49) | 0.480 |
| Insurance Status | Uninsured | REF |  |
|  | Private Insurance | 0.97 (0.71-1.33) | 0.844 |
|  | Medicaid | 1.19 (0.76-1.86) | 0.456 |
|  | Medicare | 0.80 (0.56-1.14) | 0.217 |
| Education Level | >21% | REF |  |
|  | 13%-20.9% | 1.04 (0.81-1.33) | 0.744 |
|  | 7%-12.9% | 1.19 (0.93-1.52) | 0.170 |
|  | <7% | 1.18 (0.89-1.58) | 0.248 |
| Medical Income | </=$47,999 | REF |  |
|  | $48,000-$62,999 | 0.85 (0.70-1.04) | 0.112 |
|  | $63,000 + | 0.70 (0.56-0.87) | 0.002 |
| Residence | Metro | REF |  |
|  | Urban | 0.86 (0.68-1.08) | 0.192 |
|  | Rural | 0.95 (0.66-1.35) | 0.770 |
| Surgery Type | Major Hepatectomy | REF |  |
|  | 36 | 0.75 (0.52-1.08) | 0.121 |
|  | 37 | 0.68 (0.47-0.97) | 0.034 |
|  | 38 | 0.62 (0.35-1.10) | 0.102 |
|  | Extended Major Hepatectomy | 0.69 (0.49-0.97) | 0.031 |
| Tumour Grade | Well | REF |  |
|  | Moderate | 1.39 (1.04-1.86) | 0.026 |
|  | Poor | 1.87 (1.38-2.52) | <0.001 |
|  | Anaplastic | 1.38 (1.00-1.92) | 0.053 |
| AJCC Pathological T Stage | pTx | REF |  |
|  | pT1 | 0.71 (0.56-0.89) | 0.004 |
|  | pT2 | 0.83 (0.68-1.01) | 0.069 |
|  | pT3 | 1.20 (0.96-1.50) | 0.107 |
|  | pT4 | 1.38 (1.05-1.82) | 0.023 |
| AJCC Pathological N Stage | N0 | REF |  |
|  | N1 | 1.60 (1.33-1.93) | <0.001 |
|  | N2 | 1.83 (1.28-2.61) | 0.001 |
|  | N3 | 2.04 (1.23-3.39) | 0.006 |
|  | Nx | 1.11 (0.93-1.31) | 0.253 |
| Margin Status | Negative | REF |  |
|  | Positive | 1.43 (1.22-1.67) | <0.001 |
| Lymphovascular Invasion | Absent | REF |  |
|  | Present | 1.35 (1.12-1.63) | 0.002 |
| Adjuvant Radiotherapy | No | REF |  |
|  | Yes | 0.84 (0.71-0.99) | 0.038 |
| Chemotherapy treatment | Neoadjuvant | REF |  |
|  | Adjuvant | 1.19 (0.99-1.45) | 0.068 |

Supplementary Table 4. Characteristics of patients undergoing SA, NAC or AC for perihilar cholangiocarcinoma.

|  |  | Surgery alone | Neoadjuvant | Adjuvant | P value |
| --- | --- | --- | --- | --- | --- |
| Facility Type | Community | 207 (22.0) | 24 (19.5) | 226 (27.7) | 0.001 |
|  | Academic | 621 (66.1) | 73 (59.3) | 475 (58.2) |  |
|  | Others | 112 (11.9) | 26 (21.1) | 115 (14.1) |  |
| Facility Location | Northeast | 196 (20.9) | 17 (13.8) | 187 (22.9) | 0.201 |
|  | South | 277 (29.5) | 32 (26.0) | 235 (28.8) |  |
|  | Midwest | 214 (22.8) | 37 (30.1) | 189 (23.2) |  |
|  | West | 253 (26.9) | 37 (30.1) | 205 (25.1) |  |
| Hospital Distance | <12.5 miles | 298 (31.7) | 35 (28.5) | 382 (46.8) | <0.001 |
|  | 12.5-49.9 miles | 316 (33.6) | 40 (32.5) | 289 (35.4) |  |
|  | >/=50 miles | 326 (34.7) | 48 (39.0) | 145 (17.8) |  |
| Year of Diagnosis | 2006-2007 | 0 (0.0) | 0 (0.0) | 0 (0.0) | 0.060 |
|  | 2008-2009 | 0 (0.0) | 0 (0.0) | 0 (0.0) |  |
|  | 2010-2011 | 257 (27.3) | 32 (26.0) | 232 (28.4) |  |
|  | 2012-2013 | 293 (31.2) | 29 (23.6) | 210 (25.7) |  |
|  | 2014-2016 | 390 (41.5) | 62 (50.4) | 374 (45.8) |  |
| Age at Diagnosis, years | 36-50 | 69 (7.3) | 22 (17.9) | 101 (12.4) | <0.001 |
|  | 51-65 | 263 (28.0) | 56 (45.5) | 330 (40.4) |  |
|  | 66-80 | 491 (52.2) | 41 (33.3) | 361 (44.2) |  |
|  | 80+ | 114 (12.1) | 2 (1.6) | 24 (2.9) |  |
|  | (Missing) | 3 (0.3) | 2 (1.6) | 0 (0.0) |  |
| Sex | Male | 590 (62.8) | 71 (57.7) | 492 (60.3) | 0.394 |
|  | Female | 350 (37.2) | 52 (42.3) | 324 (39.7) |  |
| Race | White | 794 (84.5) | 113 (91.9) | 671 (82.2) | 0.040 |
|  | Black | 55 (5.9) | 6 (4.9) | 65 (8.0) |  |
|  | Other | 91 (9.7) | 4 (3.3) | 80 (9.8) |  |
| CDCC Score | 0-1 | 867 (92.2) | 113 (91.9) | 772 (94.6) | 0.117 |
|  | 2+ | 73 (7.8) | 10 (8.1) | 44 (5.4) |  |
| Insurance Status | Uninsured | 63 (6.7) | 8 (6.5) | 48 (5.9) | <0.001 |
|  | Private Insurance | 303 (32.2) | 54 (43.9) | 374 (45.8) |  |
|  | Medicaid | 50 (5.3) | 6 (4.9) | 50 (6.1) |  |
|  | Medicare | 524 (55.7) | 55 (44.7) | 344 (42.2) |  |
| Education Level | >21% | 194 (20.6) | 9 (7.3) | 122 (15.0) | 0.001 |
|  | 13%-20.9% | 232 (24.7) | 30 (24.4) | 203 (24.9) |  |
|  | 7%-12.9% | 291 (31.0) | 48 (39.0) | 256 (31.4) |  |
|  | <7% | 223 (23.7) | 36 (29.3) | 235 (28.8) |  |
| Medical Income | </=$47,999 | 357 (38.0) | 40 (32.5) | 275 (33.7) | 0.075 |
|  | $48,000-$62,999 | 284 (30.2) | 34 (27.6) | 233 (28.6) |  |
|  | $63,000 + | 299 (31.8) | 49 (39.8) | 308 (37.7) |  |
| Residence | Metro | 766 (81.5) | 108 (87.8) | 695 (85.2) | 0.112 |
|  | Urban | 127 (13.5) | 9 (7.3) | 92 (11.3) |  |
|  | Rural | 47 (5.0) | 6 (4.9) | 29 (3.6) |  |
| Surgery Type | Major Hepatectomy | 597 (63.5) | 71 (57.7) | 492 (60.3) | 0.245 |
|  | Extended Major Hepatectomy | 343 (36.5) | 52 (42.3) | 324 (39.7) |  |
| Tumour Grade | Well | 140 (14.9) | 13 (10.6) | 99 (12.1) | <0.001 |
|  | Moderate | 402 (42.8) | 25 (20.3) | 404 (49.5) |  |
|  | Poor | 248 (26.4) | 16 (13.0) | 212 (26.0) |  |
|  | Anaplastic | 150 (16.0) | 69 (56.1) | 101 (12.4) |  |
| AJCC Pathological T Stage | pTx | 86 (9.1) | 28 (22.8) | 52 (6.4) | <0.001 |
|  | pT1 | 164 (17.4) | 22 (17.9) | 41 (5.0) |  |
|  | pT2 | 451 (48.0) | 43 (35.0) | 441 (54.0) |  |
|  | pT3 | 197 (21.0) | 17 (13.8) | 236 (28.9) |  |
|  | pT4 | 42 (4.5) | 13 (10.6) | 46 (5.6) |  |
| AJCC Pathological N Stage | N0 | 493 (52.4) | 69 (56.1) | 320 (39.2) | <0.001 |
|  | N1 | 239 (25.4) | 16 (13.0) | 302 (37.0) |  |
|  | N2 | 39 (4.1) | 6 (4.9) | 64 (7.8) |  |
|  | N3 | 14 (1.5) | 1 (0.8) | 27 (3.3) |  |
|  | Nx | 155 (16.5) | 31 (25.2) | 103 (12.6) |  |
| Margin Status | Negative | 651 (69.3) | 104 (84.6) | 491 (60.2) | <0.001 |
|  | Positive | 289 (30.7) | 19 (15.4) | 325 (39.8) |  |
| Lymphovascular Invasion | Absent | 637 (67.8) | 100 (81.3) | 505 (61.9) | <0.001 |
|  | Present | 303 (32.2) | 23 (18.7) | 311 (38.1) |  |
| Adjuvant Radiotherapy | No | 905 (96.3) | 117 (95.1) | 337 (41.3) | <0.001 |
|  | Yes | 35 (3.7) | 6 (4.9) | 479 (58.7) |  |

Supplementary Table 5. Characteristics among patient undergoing NAC and AC for perihilar cholangiocarcinoma.

|  |  | Neoadjuvant | Adjuvant | P value |
| --- | --- | --- | --- | --- |
| Facility Type | Community | 24 (19.5) | 226 (27.7) | 0.044 |
|  | Academic | 73 (59.3) | 475 (58.2) |  |
|  | Others | 26 (21.1) | 115 (14.1) |  |
| Facility Location | Northeast | 17 (13.8) | 187 (22.9) | 0.057 |
|  | South | 32 (26.0) | 235 (28.8) |  |
|  | Midwest | 37 (30.1) | 189 (23.2) |  |
|  | West | 37 (30.1) | 205 (25.1) |  |
| Hospital Distance | <12.5 miles | 35 (28.5) | 382 (46.8) | <0.001 |
|  | 12.5-49.9 miles | 40 (32.5) | 289 (35.4) |  |
|  | >/=50 miles | 48 (39.0) | 145 (17.8) |  |
| Year of Diagnosis | 2006-2007 | 0 (0.0) | 0 (0.0) | 0.638 |
|  | 2008-2009 | 0 (0.0) | 0 (0.0) |  |
|  | 2010-2011 | 32 (26.0) | 232 (28.4) |  |
|  | 2012-2013 | 29 (23.6) | 210 (25.7) |  |
|  | 2014-2016 | 62 (50.4) | 374 (45.8) |  |
| Age at Diagnosis, years | 36-50 | 22 (17.9) | 101 (12.4) | 0.077 |
|  | 51-65 | 56 (45.5) | 330 (40.4) |  |
|  | 66-80 | 41 (33.3) | 361 (44.2) |  |
|  | 80+ | 2 (1.6) | 24 (2.9) |  |
|  | (Missing) | 2 (1.6) | 0 (0.0) |  |
| Sex | Male | 71 (57.7) | 492 (60.3) | 0.657 |
|  | Female | 52 (42.3) | 324 (39.7) |  |
| Race | White | 113 (91.9) | 671 (82.2) | 0.022 |
|  | Black | 6 (4.9) | 65 (8.0) |  |
|  | Other | 4 (3.3) | 80 (9.8) |  |
| CDCC Score | 0-1 | 113 (91.9) | 772 (94.6) | 0.313 |
|  | 2+ | 10 (8.1) | 44 (5.4) |  |
| Insurance Status | Uninsured | 8 (6.5) | 48 (5.9) | 0.896 |
|  | Private Insurance | 54 (43.9) | 374 (45.8) |  |
|  | Medicaid | 6 (4.9) | 50 (6.1) |  |
|  | Medicare | 55 (44.7) | 344 (42.2) |  |
| Education Level | >21% | 9 (7.3) | 122 (15.0) | 0.093 |
|  | 13%-20.9% | 30 (24.4) | 203 (24.9) |  |
|  | 7%-12.9% | 48 (39.0) | 256 (31.4) |  |
|  | <7% | 36 (29.3) | 235 (28.8) |  |
| Medical Income | </=$47,999 | 40 (32.5) | 275 (33.7) | 0.905 |
|  | $48,000-$62,999 | 34 (27.6) | 233 (28.6) |  |
|  | $63,000 + | 49 (39.8) | 308 (37.7) |  |
| Residence | Metro | 108 (87.8) | 695 (85.2) | 0.342 |
|  | Urban | 9 (7.3) | 92 (11.3) |  |
|  | Rural | 6 (4.9) | 29 (3.6) |  |
| Surgery Type | Major Hepatectomy | 71 (57.7) | 492 (60.3) | 0.657 |
|  | Extended Major Hepatectomy | 52 (42.3) | 324 (39.7) |  |
| Tumour Grade | Well | 13 (10.6) | 99 (12.1) | <0.001 |
|  | Moderate | 25 (20.3) | 404 (49.5) |  |
|  | Poor | 16 (13.0) | 212 (26.0) |  |
|  | Anaplastic | 69 (56.1) | 101 (12.4) |  |
| AJCC Pathological T Stage | pTx | 28 (22.8) | 52 (6.4) | <0.001 |
|  | pT1 | 22 (17.9) | 41 (5.0) |  |
|  | pT2 | 43 (35.0) | 441 (54.0) |  |
|  | pT3 | 17 (13.8) | 236 (28.9) |  |
|  | pT4 | 13 (10.6) | 46 (5.6) |  |
| AJCC Pathological N Stage | N0 | 69 (56.1) | 320 (39.2) | <0.001 |
|  | N1 | 16 (13.0) | 302 (37.0) |  |
|  | N2 | 6 (4.9) | 64 (7.8) |  |
|  | N3 | 1 (0.8) | 27 (3.3) |  |
|  | Nx | 31 (25.2) | 103 (12.6) |  |
| Margin Status | Negative | 104 (84.6) | 491 (60.2) | <0.001 |
|  | Positive | 19 (15.4) | 325 (39.8) |  |
| Lymphovascular Invasion | Absent | 100 (81.3) | 505 (61.9) | <0.001 |
|  | Present | 23 (18.7) | 311 (38.1) |  |
| Adjuvant Radiotherapy | No | 117 (95.1) | 337 (41.3) | <0.001 |
|  | Yes | 6 (4.9) | 479 (58.7) |  |

Supplementary Table 6. Cox regression multivariable model on overall survival for perihilar cholangiocarcinoma

|  |  | Hazard ratio (CI_95%_) | P value |
| --- | --- | --- | --- |
| Facility Type | Community | REF |  |
|  | Academic | 0.75 (0.59-0.96) | 0.020 |
|  | Others | 0.92 (0.67-1.26) | 0.587 |
| Facility Location | Northeast | REF |  |
|  | South | 1.03 (0.77-1.38) | 0.838 |
|  | Midwest | 1.22 (0.91-1.63) | 0.186 |
|  | West | 0.89 (0.65-1.20) | 0.442 |
| Hospital Distance | <12.5 miles | REF |  |
|  | 12.5-49.9 miles | 0.95 (0.76-1.19) | 0.685 |
|  | >/=50 miles | 0.79 (0.59-1.04) | 0.095 |
| Year of Diagnosis | 2006-2007 | REF |  |
|  | 2008-2009 | NA | NA |
|  | 2010-2011 | 1.11 (0.86-1.43) | 0.417 |
|  | 2012-2013 | 1.09 (0.85-1.39) | 0.503 |
|  | 2014-2016 | NA | NA |
| Age at Diagnosis, years | 36-50 | REF |  |
|  | 51-65 | 1.33 (0.95-1.85) | 0.092 |
|  | 66-80 | 1.32 (0.88-1.98) | 0.180 |
|  | 80+ | 3.30 (1.78-6.11) | <0.001 |
| Sex | Male | REF |  |
|  | Female | 1.14 (0.94-1.38) | 0.195 |
| Race | White | REF |  |
|  | Black | 0.92 (0.64-1.31) | 0.636 |
|  | Other | 1.64 (1.15-2.34) | 0.006 |
| CDCC Score | 0-1 | REF |  |
|  | 2+ | 1.24 (0.85-1.83) | 0.265 |
| Insurance Status | Uninsured | REF |  |
|  | Private Insurance | 0.99 (0.64-1.54) | 0.969 |
|  | Medicaid | 0.95 (0.52-1.71) | 0.859 |
|  | Medicare | 1.13 (0.70-1.81) | 0.625 |
| Education Level | >21% | REF |  |
|  | 13%-20.9% | 0.95 (0.68-1.33) | 0.763 |
|  | 7%-12.9% | 1.04 (0.74-1.46) | 0.836 |
|  | <7% | 1.15 (0.78-1.70) | 0.480 |
| Medical Income | </=$47,999 | REF |  |
|  | $48,000-$62,999 | 0.99 (0.77-1.28) | 0.963 |
|  | $63,000 + | 0.79 (0.58-1.07) | 0.131 |
| Residence | Metro | REF |  |
|  | Urban | 1.12 (0.80-1.57) | 0.494 |
|  | Rural | 1.26 (0.77-2.04) | 0.355 |
| Surgery Type | Major Hepatectomy | REF |  |
|  | Extended Major Hepatectomy | 1.22 (1.01-1.49) | 0.042 |
| Tumour Grade | Well | REF |  |
|  | Moderate | 1.35 (0.96-1.91) | 0.085 |
|  | Poor | 1.53 (1.06-2.20) | 0.024 |
|  | Anaplastic | 1.65 (1.13-2.42) | 0.010 |
| AJCC Pathological T Stage | pTx | REF |  |
|  | pT1 | 0.81 (0.47-1.40) | 0.453 |
|  | pT2 | 1.15 (0.78-1.68) | 0.476 |
|  | pT3 | 1.13 (0.75-1.70) | 0.555 |
|  | pT4 | 1.19 (0.73-1.93) | 0.485 |
| AJCC Pathological N Stage | N0 | REF |  |
|  | N1 | 1.56 (1.23-1.98) | <0.001 |
|  | N2 | 1.56 (1.05-2.33) | 0.028 |
|  | N3 | 3.42 (2.12-5.54) | <0.001 |
|  | Nx | 1.65 (1.23-2.22) | 0.001 |
| Margin Status | Negative | REF |  |
|  | Positive | 1.58 (1.29-1.94) | <0.001 |
| Lymphovascular Invasion | Absent | REF |  |
|  | Present | 1.21 (0.97-1.52) | 0.085 |
| Adjuvant Radiotherapy | No | REF |  |
|  | Yes | 0.80 (0.65-0.99) | 0.038 |
| Chemotherapy treatment | Neoadjuvant | REF |  |
|  | Adjuvant | 0.83 (0.59-1.19) | 0.311 |

Supplementary Table 7. Characteristics of patients undergoing SA, NAC or AC for distal cholangiocarcinoma.

|  |  | Surgery alone | Neoadjuvant | Adjuvant | P value |
| --- | --- | --- | --- | --- | --- |
| Facility Type | Community | 414 (21.2) | 45 (16.6) | 407 (26.5) | <0.001 |
|  | Academic | 1250 (64.0) | 171 (63.1) | 906 (59.0) |  |
|  | Others | 289 (14.8) | 55 (20.3) | 223 (14.5) |  |
| Facility Location | Northeast | 404 (20.7) | 33 (12.2) | 402 (26.2) | <0.001 |
|  | South | 669 (34.3) | 85 (31.4) | 488 (31.8) |  |
|  | Midwest | 530 (27.1) | 86 (31.7) | 357 (23.2) |  |
|  | West | 350 (17.9) | 67 (24.7) | 289 (18.8) |  |
| Hospital Distance | <12.5 miles | 657 (33.6) | 73 (26.9) | 693 (45.1) | <0.001 |
|  | 12.5-49.9 miles | 654 (33.5) | 89 (32.8) | 533 (34.7) |  |
|  | >/=50 miles | 642 (32.9) | 109 (40.2) | 310 (20.2) |  |
| Year of Diagnosis | 2006-2007 | 335 (17.2) | 33 (12.2) | 155 (10.1) | <0.001 |
|  | 2008-2009 | 362 (18.5) | 40 (14.8) | 263 (17.1) |  |
|  | 2010-2011 | 334 (17.1) | 44 (16.2) | 271 (17.6) |  |
|  | 2012-2013 | 345 (17.7) | 62 (22.9) | 299 (19.5) |  |
|  | 2014-2016 | 577 (29.5) | 92 (33.9) | 548 (35.7) |  |
| Age at Diagnosis, years | 36-50 | 148 (7.6) | 60 (22.1) | 199 (13.0) | <0.001 |
|  | 51-65 | 634 (32.5) | 126 (46.5) | 644 (41.9) |  |
|  | 66-80 | 989 (50.6) | 77 (28.4) | 640 (41.7) |  |
|  | 80+ | 179 (9.2) | 5 (1.8) | 49 (3.2) |  |
|  | (Missing) | 3 (0.2) | 3 (1.1) | 4 (0.3) |  |
| Sex | Male | 1141 (58.4) | 158 (58.3) | 915 (59.6) | 0.776 |
|  | Female | 812 (41.6) | 113 (41.7) | 621 (40.4) |  |
| Race | White | 1652 (84.6) | 245 (90.4) | 1267 (82.5) | 0.016 |
|  | Black | 136 (7.0) | 13 (4.8) | 131 (8.5) |  |
|  | Other | 165 (8.4) | 13 (4.8) | 138 (9.0) |  |
| CDCC Score | 0-1 | 1748 (89.5) | 249 (91.9) | 1442 (93.9) | <0.001 |
|  | 2+ | 205 (10.5) | 22 (8.1) | 94 (6.1) |  |
| Insurance Status | Uninsured | 126 (6.5) | 17 (6.3) | 87 (5.7) | <0.001 |
|  | Private Insurance | 650 (33.3) | 146 (53.9) | 723 (47.1) |  |
|  | Medicaid | 112 (5.7) | 11 (4.1) | 88 (5.7) |  |
|  | Medicare | 1065 (54.5) | 97 (35.8) | 638 (41.5) |  |
| Education Level | >21% | 330 (16.9) | 29 (10.7) | 237 (15.4) | 0.012 |
|  | 13%-20.9% | 481 (24.6) | 62 (22.9) | 338 (22.0) |  |
|  | 7%-12.9% | 656 (33.6) | 95 (35.1) | 514 (33.5) |  |
|  | <7% | 486 (24.9) | 85 (31.4) | 447 (29.1) |  |
| Medical Income | </=$47,999 | 784 (40.1) | 97 (35.8) | 497 (32.4) | <0.001 |
|  | $48,000-$62,999 | 519 (26.6) | 77 (28.4) | 435 (28.3) |  |
|  | $63,000 + | 650 (33.3) | 97 (35.8) | 604 (39.3) |  |
| Residence | Metro | 1581 (81.0) | 217 (80.1) | 1264 (82.3) | 0.402 |
|  | Urban | 273 (14.0) | 44 (16.2) | 211 (13.7) |  |
|  | Rural | 99 (5.1) | 10 (3.7) | 61 (4.0) |  |
| Surgery Type | Major Hepatectomy | 837 (42.9) | 104 (38.4) | 758 (49.3) | <0.001 |
|  | 36 | 154 (7.9) | 12 (4.4) | 45 (2.9) |  |
|  | 37 | 164 (8.4) | 13 (4.8) | 64 (4.2) |  |
|  | 38 | 9 (0.5) | 0 (0.0) | 3 (0.2) |  |
|  | Extended Major Hepatectomy | 789 (40.4) | 142 (52.4) | 666 (43.4) |  |
| Tumour Grade | Well | 252 (12.9) | 26 (9.6) | 123 (8.0) | <0.001 |
|  | Moderate | 887 (45.4) | 75 (27.7) | 714 (46.5) |  |
|  | Poor | 532 (27.2) | 53 (19.6) | 493 (32.1) |  |
|  | Anaplastic | 282 (14.4) | 117 (43.2) | 206 (13.4) |  |
| AJCC Pathological T Stage | pTx | 638 (32.7) | 103 (38.0) | 351 (22.9) | <0.001 |
|  | pT1 | 281 (14.4) | 58 (21.4) | 73 (4.8) |  |
|  | pT2 | 498 (25.5) | 47 (17.3) | 461 (30.0) |  |
|  | pT3 | 465 (23.8) | 47 (17.3) | 579 (37.7) |  |
|  | pT4 | 71 (3.6) | 16 (5.9) | 72 (4.7) |  |
| AJCC Pathological N Stage | N0 | 930 (47.6) | 136 (50.2) | 599 (39.0) | <0.001 |
|  | N1 | 407 (20.8) | 45 (16.6) | 539 (35.1) |  |
|  | N2 | 87 (4.5) | 7 (2.6) | 119 (7.7) |  |
|  | N3 | 34 (1.7) | 4 (1.5) | 66 (4.3) |  |
|  | Nx | 495 (25.3) | 79 (29.2) | 213 (13.9) |  |
| Margin Status | Negative | 1517 (77.7) | 219 (80.8) | 1033 (67.3) | <0.001 |
|  | Positive | 436 (22.3) | 52 (19.2) | 503 (32.7) |  |
| Lymphovascular Invasion | Absent | 1592 (81.5) | 234 (86.3) | 1086 (70.7) | <0.001 |
|  | Present | 361 (18.5) | 37 (13.7) | 450 (29.3) |  |
| Adjuvant Radiotherapy | No | 1882 (96.4) | 249 (91.9) | 722 (47.0) | <0.001 |
|  | Yes | 71 (3.6) | 22 (8.1) | 814 (53.0) |  |

Supplementary Table 8. Characteristics among patient undergoing NAC and AC for distal cholangiocarcinoma.

|  |  | Neoadjuvant | Adjuvant | p |
| --- | --- | --- | --- | --- |
| Facility Type | Community | 45 (16.6) | 407 (26.5) | 0.001 |
|  | Academic | 171 (63.1) | 906 (59.0) |  |
|  | Others | 55 (20.3) | 223 (14.5) |  |
| Facility Location | Northeast | 33 (12.2) | 402 (26.2) | <0.001 |
|  | South | 85 (31.4) | 488 (31.8) |  |
|  | Midwest | 86 (31.7) | 357 (23.2) |  |
|  | West | 67 (24.7) | 289 (18.8) |  |
| Hospital Distance | <12.5 miles | 73 (26.9) | 693 (45.1) | <0.001 |
|  | 12.5-49.9 miles | 89 (32.8) | 533 (34.7) |  |
|  | >/=50 miles | 109 (40.2) | 310 (20.2) |  |
| Year of Diagnosis | 2006-2007 | 33 (12.2) | 155 (10.1) | 0.474 |
|  | 2008-2009 | 40 (14.8) | 263 (17.1) |  |
|  | 2010-2011 | 44 (16.2) | 271 (17.6) |  |
|  | 2012-2013 | 62 (22.9) | 299 (19.5) |  |
|  | 2014-2016 | 92 (33.9) | 548 (35.7) |  |
| Age at Diagnosis, years | 36-50 | 60 (22.1) | 199 (13.0) | <0.001 |
|  | 51-65 | 126 (46.5) | 644 (41.9) |  |
|  | 66-80 | 77 (28.4) | 640 (41.7) |  |
|  | 80+ | 5 (1.8) | 49 (3.2) |  |
|  | (Missing) | 3 (1.1) | 4 (0.3) |  |
| Sex | Male | 158 (58.3) | 915 (59.6) | 0.745 |
|  | Female | 113 (41.7) | 621 (40.4) |  |
| Race | White | 245 (90.4) | 1267 (82.5) | 0.005 |
|  | Black | 13 (4.8) | 131 (8.5) |  |
|  | Other | 13 (4.8) | 138 (9.0) |  |
| CDCC Score | 0-1 | 249 (91.9) | 1442 (93.9) | 0.270 |
|  | 2+ | 22 (8.1) | 94 (6.1) |  |
| Insurance Status | Uninsured | 17 (6.3) | 87 (5.7) | 0.144 |
|  | Private Insurance | 146 (53.9) | 723 (47.1) |  |
|  | Medicaid | 11 (4.1) | 88 (5.7) |  |
|  | Medicare | 97 (35.8) | 638 (41.5) |  |
| Education Level | >21% | 29 (10.7) | 237 (15.4) | 0.245 |
|  | 13%-20.9% | 62 (22.9) | 338 (22.0) |  |
|  | 7%-12.9% | 95 (35.1) | 514 (33.5) |  |
|  | <7% | 85 (31.4) | 447 (29.1) |  |
| Medical Income | </=$47,999 | 97 (35.8) | 497 (32.4) | 0.457 |
|  | $48,000-$62,999 | 77 (28.4) | 435 (28.3) |  |
|  | $63,000 + | 97 (35.8) | 604 (39.3) |  |
| Residence | Metro | 217 (80.1) | 1264 (82.3) | 0.548 |
|  | Urban | 44 (16.2) | 211 (13.7) |  |
|  | Rural | 10 (3.7) | 61 (4.0) |  |
| Surgery Type | Major Hepatectomy | 104 (38.4) | 758 (49.3) | 0.015 |
|  | 36 | 12 (4.4) | 45 (2.9) |  |
|  | 37 | 13 (4.8) | 64 (4.2) |  |
|  | 38 | 0 (0.0) | 3 (0.2) |  |
|  | Extended Major Hepatectomy | 142 (52.4) | 666 (43.4) |  |
| Tumour Grade | Well | 26 (9.6) | 123 (8.0) | <0.001 |
|  | Moderate | 75 (27.7) | 714 (46.5) |  |
|  | Poor | 53 (19.6) | 493 (32.1) |  |
|  | Anaplastic | 117 (43.2) | 206 (13.4) |  |
| AJCC Pathological T Stage | pTx | 103 (38.0) | 351 (22.9) | <0.001 |
|  | pT1 | 58 (21.4) | 73 (4.8) |  |
|  | pT2 | 47 (17.3) | 461 (30.0) |  |
|  | pT3 | 47 (17.3) | 579 (37.7) |  |
|  | pT4 | 16 (5.9) | 72 (4.7) |  |
| AJCC Pathological N Stage | N0 | 136 (50.2) | 599 (39.0) | <0.001 |
|  | N1 | 45 (16.6) | 539 (35.1) |  |
|  | N2 | 7 (2.6) | 119 (7.7) |  |
|  | N3 | 4 (1.5) | 66 (4.3) |  |
|  | Nx | 79 (29.2) | 213 (13.9) |  |
| Margin Status | Negative | 219 (80.8) | 1033 (67.3) | <0.001 |
|  | Positive | 52 (19.2) | 503 (32.7) |  |
| Lymphovascular Invasion | Absent | 234 (86.3) | 1086 (70.7) | <0.001 |
|  | Present | 37 (13.7) | 450 (29.3) |  |
| Adjuvant Radiotherapy | No | 249 (91.9) | 722 (47.0) | <0.001 |
|  | Yes | 22 (8.1) | 814 (53.0) |  |

Supplementary Table 9. Cox regression multivariable model on overall survival for distal cholangiocarcinoma

|  |  | Hazard ratio (CI_95%_) | P value |
| --- | --- | --- | --- |
| Facility Type | Community | REF |  |
|  | Academic | 0.86 (0.73-1.01) | 0.059 |
|  | Others | 1.05 (0.85-1.30) | 0.633 |
| Facility Location | Northeast | REF |  |
|  | South | 0.98 (0.82-1.18) | 0.839 |
|  | Midwest | 1.04 (0.86-1.26) | 0.677 |
|  | West | 0.92 (0.75-1.14) | 0.447 |
| Hospital Distance | <12.5 miles | REF |  |
|  | 12.5-49.9 miles | 1.10 (0.95-1.28) | 0.192 |
|  | >/=50 miles | 0.95 (0.79-1.15) | 0.614 |
| Year of Diagnosis | 2006-2007 | REF |  |
|  | 2008-2009 | 0.98 (0.79-1.21) | 0.852 |
|  | 2010-2011 | 0.71 (0.57-0.89) | 0.004 |
|  | 2012-2013 | 0.73 (0.58-0.91) | 0.006 |
|  | 2014-2016 | 0.71 (0.56-0.91) | 0.007 |
| Age at Diagnosis, years | 36-50 | REF |  |
|  | 51-65 | 1.18 (0.96-1.43) | 0.112 |
|  | 66-80 | 1.34 (1.05-1.70) | 0.020 |
|  | 80+ | 2.27 (1.52-3.38) | <0.001 |
| Sex | Male | REF |  |
|  | Female | 0.89 (0.78-1.01) | 0.072 |
| Race | White | REF |  |
|  | Black | 0.99 (0.77-1.26) | 0.910 |
|  | Other | 1.09 (0.86-1.39) | 0.477 |
| CDCC Score | 0-1 | REF |  |
|  | 2+ | 1.32 (1.02-1.71) | 0.035 |
| Insurance Status | Uninsured | REF |  |
|  | Private Insurance | 0.86 (0.65-1.14) | 0.308 |
|  | Medicaid | 0.93 (0.63-1.37) | 0.710 |
|  | Medicare | 0.88 (0.64-1.19) | 0.394 |
| Education Level | >21% | REF |  |
|  | 13%-20.9% | 0.99 (0.79-1.23) | 0.915 |
|  | 7%-12.9% | 0.96 (0.77-1.21) | 0.750 |
|  | <7% | 1.00 (0.77-1.29) | 0.978 |
| Medical Income | </=$47,999 | REF |  |
|  | $48,000-$62,999 | 0.96 (0.80-1.15) | 0.682 |
|  | $63,000 + | 0.81 (0.66-1.00) | 0.054 |
| Residence | Metro | REF |  |
|  | Urban | 0.92 (0.75-1.12) | 0.400 |
|  | Rural | 0.86 (0.61-1.21) | 0.381 |
| Surgery Type | Major Hepatectomy | REF |  |
|  | 36 | 1.06 (0.74-1.52) | 0.746 |
|  | 37 | 1.07 (0.77-1.49) | 0.689 |
|  | 38 | 0.42 (0.06-3.05) | 0.391 |
|  | Extended Major Hepatectomy | 1.01 (0.88-1.15) | 0.909 |
| Tumour Grade | Well | REF |  |
|  | Moderate | 1.35 (1.04-1.74) | 0.022 |
|  | Poor | 1.62 (1.25-2.11) | <0.001 |
|  | Anaplastic | 1.59 (1.19-2.11) | 0.002 |
| AJCC Pathological T Stage | pTx | REF |  |
|  | pT1 | 0.82 (0.61-1.11) | 0.201 |
|  | pT2 | 1.03 (0.85-1.25) | 0.729 |
|  | pT3 | 1.19 (0.98-1.44) | 0.073 |
|  | pT4 | 1.30 (0.97-1.75) | 0.082 |
| AJCC Pathological N Stage | N0 | REF |  |
|  | N1 | 1.60 (1.37-1.87) | <0.001 |
|  | N2 | 1.81 (1.40-2.34) | <0.001 |
|  | N3 | 2.66 (1.94-3.65) | <0.001 |
|  | Nx | 1.40 (1.15-1.71) | 0.001 |
| Margin Status | Negative | REF |  |
|  | Positive | 1.43 (1.24-1.64) | <0.001 |
| Lymphovascular Invasion | Absent | REF |  |
|  | Present | 1.07 (0.90-1.28) | 0.443 |
| Adjuvant Radiotherapy | No | REF |  |
|  | Yes | 0.84 (0.73-0.96) | 0.012 |
| Chemotherapy treatment | Neoadjuvant | REF |  |
|  | Adjuvant | 1.13 (0.91-1.41) | 0.264 |
